# Odds and Ends, or Hints from an Experience of Twenty Years in Dental Practice

**Published:** 1859-04

**Authors:** A. Hill


### ARTICLE V.

### *Odds and Ends, or Hints from an experience of twenty years in Dental Practice.* By A. Hill, D. D. S.

### The Eye and the Hand.

The *natural* and the *trained* eye are two things. The hand *educated*, or *uneducated*, are widely different.

It is one thing to educate the mind, and quite another to educate these organs as *instruments* of the mind. Paginini's head, without Paginini's hands, would have failed to achieve his present world-wide renown. And thus much is true of every musician.

The same instrument that responds in such a marvellous manner, when its strings are swept by a master hand, is quite tame and common-place in the hands of a novice.

It is not enough that we have the mental conception. That conception must be wrought out—embodied, crystallized. To this end, instrumentalities are necessary.

*Man* and the *tools* he works with, might well supply an inexhaustible theme.

But we leave the subject in its widest range, and confine ourselves for the present to the *eye* and the *hand* of the dentist.

Some things can only be learned from *experience*. No books, however good—no instructors however well skilled, can properly teach them. Or, if they be able to suggest, or call attention to them, they cannot be apprehended by the student, until his senses are quickened by exercise, and educated to a degree, of which he, in his novitiate, has really no conception.

Such, for example, is the exquisite sense of what we call a *practiced* eye, an eye educated to see what other eyes must always fail to discover, until they are specifically pointed out. An artist will easily detect in a picture, what non-professional observers might gaze upon for years, without ever beholding. And this too, whether it relates to the merits or defects of the same.

Lectures on *auscultation* may be of great service to show us what has, or may be done, and to *prepare* the student to *commence* his experiments to the best advantage. But what else than a *practiced* ear, can detect incipient disease, or diagnose its successive stages, by the use of the stethoscope?

That exalted sensibility which only waits on long and patient observation, and the most delicate experiment can never be gained by mere assumption. No dashing effort of the will, no *coup-de-main* can ever secure it.

Instruct a student as you may, let him study until he can rehearse all that the books contain with respect to the circulation of the blood, and the peculiarities of its pulsations through the body; yet, until the tips of his fingers are educated, and the subtle soul has learned to be instructed by them, it is in vain that he puts them upon his patient's wrist. The important distinctions between "*hard*" and "*full*"—"*soft*" and "*feeble*"—"*rapid*" and "*quick*"—"*thread-like*" and "*wiry*," &c., he can only learn at the bed-side, and by the most unremitting and scrupulous attention.

That recondite sense—that exquisite appreciation, which is wielded only by an "*expert*," is precisely that which gives the highest professional eminence, and marks the boundary between the mechanic and the artist, the man of genius and the man of routine.

That some develop this nice sense more rapidly than others, is to be conceded. But the great fact here to be insisted upon is, that *practice—manipulation—experience*, only can impart it.

We cannot leap over an unbridged chasm in pursuit of knowledge. Whether perceived or not, there must be a continuity of thought. As well might one attempt to transmit a message to Europe through the Atlantic cable, with the wires parted in mid ocean, as to try to reach a given point in science without taking the intermediate steps which lead to it.

We are aware, that what men call "*genius*," seems sometimes to scorn the ordinary rules of intellectual development, and to reach conclusions by the mere force of apparent *intuition*, while the ordinary student is plodding on through the painful drudgery of concatenated thought.

The mind is not always conscious of its own operations. It cannot always stop to analyze the process through which it reaches its conclusions. Yet there are good reasons for believing, that even in those cases, where results are attained by what we should call the *intuitional* action of the mind, it is only because the mental action is so subtle and quick, that we fail to observe the successive stages of its progress.

So that in general we may safely say, we *need* to know *some* things, in order to learn *others*, and that really, all the intervening space must be occupied before we can reach a given stage in our intellectual journey.

Every reader must be conscious of the fact of having had his own mind burdened with words and sentences unintelligible to himself, and his memory stored with phrases, having to him, no signification.

And he must be equally conscious of a time, when such words or phrases have opened upon his intellect with a strange and beautiful significance. These words, hitherto so cabalistic and unmeaning, now corruscate with such a light as to irradiate the whole mind so beautifully as to impart an indescribable charm to the mental process by which their new glory has been inaugurated.

In mental development, ideasare proportioned to capacity. The mind can only embrace those ideas which fall within the scope of its development. Words being the natural vehicles of thought, can only be comprehended by a mind sufficiently mature to receive them. To a well disciplined mind, language has a meaning and a power, to which others are strangers. How strangely, for instance, the terse language and compact logic of Butler's Analogy, contrasts with the insane and inane jargon of "Joe Smith," or Andrew Jackson Davis!

How wide the pictures which are intended to adorn our public galleries, in some instances vary, from the true standard of excellence! And how striking the contrast to those who have an eye to perceive it, between the mass of them, and those exquisite productions of Church or Rembrandt.

Equally great, to an eye capable of seeing, is the difference between those dental operations which mark the skill of a master on the one hand, and the noisy pretender on the other, whose only claim to patronage is the audacity with which he puffs himself in newspaper and pamphlet.

The unpracticed eye can no more see those subtle distinctions which mark the progress of improvement in the dental operator, as he advances from one stage of progress to another beyond a given limit, than a blind man can distinguish the beautiful colors of the rainbow.

Up to a certain point, the difference is perceptible to ordinary observers. Beyond that, the educated, the practiced eye alone, is competent to perceive.

No fault of natural vision is here pretended, but the want of that peculiar training of the perceptive faculties, which *experimental* discipline alone can give.

It is the Indian hunter, by long experience and familiarity in the chase, that is enabled to track his foe, where ordinary eyes can see no sign of foot-print. Leaves wet with the morniug dew—twigs bent or broken—the moist earth disturbed in the slightest manner by the foot of his enemy—all these, and more, are noticed by his skillful eye, and detected by a perception sharpened by practice.

Sailors will discover objects at sea, where a gaping landsman can see nothing but sky and ocean.

And thus it is, that all the faculties of man grow sharp and keen by use, and all the senses become exalted by careful cultivation.

Instances are by no means rare, where the more obscure sense of *smell* becomes important to detect disease, when the other senses fail to make the discovery. Physicians sometimes are enabled to diagnose disease by *smell alone*, under peculiar circumstances. The following instance will illustrate this.

A medical acquaintance of the writer was called to see an adult patient whose disease had baffled his attendants for some time. His physician was greatly puzzled with the case, and was unable to decide as to the nature of his ailment. Meantime the patient grew worse—the friends became alarmed and desired counsel. Another physician was called, but still no rational diagnosis could be found. The third was sent for, and on entering the room of the sick man, exclaimed, "*the man has got the measles.*" When as yet there was no sign of eruption, and nothing but the effluvia to indicate that this was his trouble. The man was raving with insanity when thus seen. Remedies in accordance with this view were administered, when the eruption soon made its appearance and the patient recovered.

The physician was asked, "how did you know that this was measles?" "I *smelt* it," was the immediate answer. And he has assured the writer, that the peculiar effluvia of that disease always makes a distinct impression upon his olfactory nerves. The same also, is frequently true of *small pox*, and other forms of eruptive diseases. Nor yet, these alone. Besides *exanthematous* diseases, other forms of fever emit a peculiar *aroma*. And the sense of *smell* may be so acute as to detect its peculiar character.

These cases are cited, simply to illustrate the important truth, upon which we design to insist, in this paper, viz. that to excel in dental operations, especially those upon the mouth and teeth, where operative dentistry finds its most appropriate sphere, the *eye*, and the *fingers must be educated*, far beyond the point of ordinary perception and sensibility.

Observe how important is this cultivated perception, as the basis of *professional opinion* merely.

What is it, that gives superior importance to one man's opinion, over that of another man, in the same profession?

Precisely this, that one man has omitted to observe certain recondite conditions and circumstances, which are absolutely essential to the formation of a correct opinion on the case; while the other, by a nicer perception and a juster appreciation of these conditions, has been enabled to challenge the confidence of his patrons.

To a person destitute of this perception, the teeth and gums of almost every mouth appear the same. No peculiarities are observed of sufficient importance to attract attention. Consequently, the basis of professional opinion is opposed to the results of practice, and the issue in such cases must inevitably lead to disappointment, disaffection, and chagrin.

In such cases, if the practitioner has sagacity enough to learn from experience, he may at length be able to discover his errors, otherwise there is no hope for him, but to go on blundering through life, doing more mischief than a hundred others can repair.

On the other hand, how different are the results! Here, a *practiced eye*, and a quick sagacious mind, is brought to the examination.

Every physical feature—every physiological indication—form—position—color—texture—temperament, and habits, are considered.

The *age*, *health* and *circumstances* of the patient are brought into the account in making up an enlightened judgment upon the case. And now the issue is not doubtful, either to foresee or predict.

Here, then, is the true basis of professional opinion. From *such* a starting point, *advice* becomes important, and is justly entitled to its reward. And now, if there be corresponding skill in manipulation—if the *tactual* sense is equal to the perception—if there be expertness in the use of instruments, and the operator is faithful to his duty, the patient will have cause for thanksgiving, as long as ability remains, to appreciate the value of the dental organism.

In *such* practice, nothing is hap-hazzard—nothing goes by guess.

The examination will be thorough and complete—the opinion mature and deliberate, and the operation skillful and satisfactory.

Every dental practitioner of several years experience, must be conscious that many operations, which in his earlier practice, he thought were superbly done, he would now blush for, if compelled to admit that he ever performed them. And why so? Simply because the progress he has made enables him to perceive, what at the time of performance he could not, that they were very imperfectly executed.

And with his present ability to perceive, he will doubtless wonder how he ever could have regarded them as good operations.

But now, the eye perceives without labor, perceives at a glance, each glaring imperfection. The *edges* were *rudely*

*filed*—the *corners* were left *sharp* and *jagged*—the *cavities* poorly prepared, and the whole thing lamentably incomplete. If no *extreme* fault subjects us to the mortification supposed, there is still enough it may be, to show a marked distinction between the present and the past.

That degree of culture, of which *any* of the senses are susceptible, is indeed a wonder. We present no new thought, when we suggest, that so marked is this susceptibility as to trench at times, the very confines of the *supernatural,* and actually raise the suspicion of some *weird* agency, which no normal development can ever manifest. But our advocacy of this culture stops short of the marvelous, only covering the truly exquisite and refined. There is a certain class of dental operations, which, from the nature of the circumstances, depend more on *feeling* than seeing, for their performance. The sense of feeling in the operator's hand, must guide him, where he cannot see. Such manipulations require great delicacy of touch, and a fine appreciation through this medium, of all the circumstances upon which a successful operation depends.

And if the culture of these powers are deficient, the operation, so far forth, must of necessity prove a failure.

In the hands of a skillful operator, the very instrument itself seems instinct with sensibility. It tells distinctly the condition of a cavity—the extent and character of caries—the loss of enamel, and the softness, hardness and other peculiarities of the subjacent bone. In short, it becomes sight to the operator where vision cannot reach, and enables him to form an opinion or achieve a result otherwise impossible.

Nor is this simple dexterity in manipulation. It is something more—something higher, a subtle sense, a keen perception, which careful culture and constant practice alone can develop.

*Dexterity* in the use of instruments will come to our aid in the packing of gold or other fillings, after the necessary preparations are made, and thus assist us to consummate, what, without this nice sense of feeling, we never could successfully complete.

The intelligent reader will not require us to point out each particular case, where this peculiar expertness is essential to success. But he will perceive at a glance, that no dental practitioner is fully qualified for his most difficult and trying duties without it.

It is most certainly *available* in all cases, but not alike *indispensable* in all.

There is a class of operations, in which a comparative bungler may be tolerably successful, but not in the cases above referred to. Here, the line of distinction is marked, and obvious, and between the two classes, there is a "great gulf fixed."

In the one case, the hand is guided as by instinct, in the other, to undertake is to fail. And hence we conclude that numberless teeth are sacrificed on the one hand, that might be made serviceable and enduring on the other.

*Stopping* teeth, and *stuffing* teeth, are two things. Multitudes can do the latter, where comparatively few are distinguished for their achievements in relation to the former.

But all such as *do* maintain a pre-eminence in this respect, illustrate in a greater or less degree, the doctrine of this paper.

Who, that ever saw the exquisite fillings of the late lamented Townsend, or was privileged to observe the rare culture and marked sensibility of the man, but will freely concede that he combined the elements of successful practice in a transcendent degree!

There are those still living—and are now enjoying the bright noon-tide of a successful and splendid practice, whose beautiful operations are the admiration of all, and the envy of some, the secret of whose success, we here set forth.

It will be the business of the future historiographer, to record their professional triumphs, when they shall have passed from the scene of their present labors, and taken their places by the side of those men, whose history is so closely identified with the enduring triumphs and masterly results of American dental practice.

